# Overexpression of *OsRRK1* Changes Leaf Morphology and Defense to Insect in Rice

**DOI:** 10.3389/fpls.2017.01783

**Published:** 2017-10-24

**Authors:** Yinhua Ma, Yan Zhao, Xinxin Shangguan, Shaojie Shi, Ya Zeng, Yan Wu, Rongzhi Chen, Aiqing You, Lili Zhu, Bo Du, Guangcun He

**Affiliations:** ^1^State Key Laboratory of Hybrid Rice, College of Life Sciences, Wuhan University, Wuhan, China; ^2^Hybrid Rice Research Center, Hubei Academy of Agricultural Sciences, Wuhan, China

**Keywords:** rice, leaf rolling, bulliform cell, RLCK, defense

## Abstract

It has been reported that the receptor-like cytoplasmic kinases (RLCKs) regulate many biological processes in plants, but only a few members have been functionally characterized. Here, we isolated a rice gene encoding *AtRRK1* homology protein kinase, *OsRRK1*, which belongs to the RLCK VI subfamily. *OsRRK1* transcript accumulated in many tissues at low to moderate levels and at high levels in leaves. Overexpression of *OsRRK1* (OE-*OsRRK1*) caused adaxial rolling and erect morphology of rice leaves. In the rolled leaves of OE-*OsRRK1* plants, both the number and the size of the bulliform cells are decreased compared to the wild-type (WT) plants. Moreover, the height, tiller number, and seed setting rate were reduced in OE-*OsRRK1* plants. In addition, the brown planthopper (BPH), a devastating pest of rice, preferred to settle on WT plants than on the OE-*OsRRK1* plants in a two-host choice test, indicating that OE-*OsRRK1* conferred an antixenosis resistance to BPH. The analysis of transcriptome sequencing demonstrated that several receptor kinases and transcription factors were differentially expressed in OE-*OsRRK1* plants and WT plants. These results indicated that *OsRRK1* may play multiple roles in the development and defense of rice, which may facilitate the breeding of novel rice varieties.

## Introduction

Increasing crop yield is a major challenge for modern agriculture (Ray et al., 2012). Regulating leaf development has been considered an effective way to achieve a breakthrough of potential yield for crops (Sinclair and Sheehy, 1999). Appropriate leaf shape is an important characteristic of the super-high-yield hybrid rice idiotype, in which the last three leaves from the top are long, erect, narrow, V-shaped (rolled), and thick (Yuan, 1997). Moderate leaf rolling in rice leads to erect leaf canopies, improves photosynthetic efficiency, accelerates dry-matter accumulation, and increases grain yield (Lang et al., 2004; Zhang et al., 2009; Zou et al., 2011). Isolation of genes controlling leaf rolling are expected to be beneficial for developing crops with the desired architecture (Zhang et al., 2009; Xu et al., 2014).

Leaf form was regulated by complicated developmental processes, including pattern formation, polarity establishment, and cell differentiation (Bowman et al., 2002; Micol and Hake, 2003; Lang et al., 2004). Bulliform cells are monocot-specific (with the exception of the Helobiae), large thin-walled, apparently empty, highly vacuolated and occur in groups between vascular bundles on the adaxial epidermis (Jane and Chiang, 1991; Itoh et al., 2005). In rice, two types of leaf rolling (adaxial rolling and abaxial rolling) have been related to the abnormal development of bulliform cells. Changes in the number, volume, and localization of bulliform cells can result in lack of osmotic pressure to support the normal form of the blade, and then, the leaf becomes rolled (Zhang et al., 2015). Generally, a leaf displays adaxial rolling when the number and size of bulliform cells are decreased in rice. For example, the mutant of a MYB transcription factor, *sll1*, has smaller and fewer bulliform cells than wild-type (WT), resulting in adaxial leaf rolling (Zhang et al., 2009). Conversely, a leaf displays abaxial rolling when the number and size of bulliform cells are increased. For example, enhanced expression of ACL1, encoding a protein with unknown conserved functional domains, causes increased bulliform cell number and abaxial leaf rolling (Li et al., 2010).

The receptor-like kinases (RLKs) play critical roles in plant development and response to stress stimuli (Gao and Xue, 2012). A typical RLK contains an extracellular receptor, a transmembrane domain and an intracellular kinase domain (Shiu and Bleecker, 2001). One of the RLK families lacking an extracellular domain has, therefore, been designated the receptor-like cytoplasmic kinases (RLCKs). Recent studies have shown that some RLCKs regulate both plant development and defense responses. In *Arabidopsis*, RLCKs are classed into 13 subfamilies according to the phylogenetic evaluation (Shiu et al., 2004). BIK1 (Botrytis-Induced Kinase 1), functions as an early-induced kinase in response to infection by *Botrytis cinerea* and is required for BRI1 (Brassinolide-Insensitive 1) mediated growth regulation through direct interaction with BRI1 (Veronese et al., 2006; Lin et al., 2013). PBL27 is an immediate downstream component of the chitin receptor CERK1 and contributes to the regulation of chitin-induced immunity in *Arabidopsis* (Shinya et al., 2014). It has been predicted that rice has a total of 379 *RLCK* genes (Vij et al., 2008). To date, few *RLCK* genes have been functionally characterized in rice. Of those that have, it has been reported that *BSR1* (broad-spectrum resistance 1) positively regulates resistance against *Xoo* (*Xanthomonas oryzae* pv. *oryzae*) and *M. oryzae* (*Magnaporthe grisea*) both in *Arabidopsis* and rice (Dubouzet et al., 2011). *OsRLCK185* and *OsRLCK55* can interact with Xoo1488, which is a Xoo effector. *OsRLCK185* also regulates PGN- and chitin-induced immunity (Yamaguchi et al., 2013). Five other *RLCK* genes, *OsRLCK102*, *OsRLCK57*, *OsRLCLK107*, *OsRLCK118*, and *OsRLCLK176* are involved in innate immunity mediated by XA21 and in development by BR signaling in rice (Yamaguchi et al., 2013; Ao et al., 2014; Wang et al., 2016; Zhou et al., 2016). Most of these reported *RLCK* genes belong to the RLCK VII subfamily and are involved in disease resistance and growth development in *Arabidopsis* and rice. However, no other members of RLCK subfamilies in rice have been reported.

In this study, we characterized the *RLCK* gene *OsRRK1*, which was interacted with *OsLecRK* (a lectin RLK) that was involved in innate immune responses and seed germination (Cheng et al., 2013), belongs to the RLCK VI subfamily and encodes an AtRRK1 (Rop-interacting receptor-like kinase 1) homologous protein kinase. Overexpression of *OsRRK1* caused rolling and erect leaves in rice plants. In addition, the degree of leaf rolling was positively correlated with the expression level of *OsRRK1*. *OsRRK1* is also involved in rice development as well as in defense against BPH. The result of transcriptome sequencing indicated that *OsRRK1* regulates plant development and defense to BPH mainly through receptor kinases, other RLCKs and transcription factors.

## Materials and Methods

### Plant Materials

In this study, the WT rice (*Oryza sativa*) cultivar used as controls in all the morphological and molecular comparisons was a *japonica* variety, Hejiang19. The template for gene amplification was derived from the cDNA of the rice *indica* variety Kasalath. All the transgenic lines including the OE and RNAi lines are in the background of Hejiang19. All experimental materials were transplanted in the experimental field at a spacing of 16.7 cm between plants within each row and 26.7 cm between rows at the Genetics Institute at Wuhan University (Wuhan, China). The plants were tended under the routine management regime.

### Yeast Two-Hybrid Assay

The two-hybrid assay was performed using the GAL4-based transcription system. The *OsRRK1* cDNA was cloned into a bait vector pGBKT7, while the *OsLecRK* cDNA was ligated into a prey vector pGADT7. Yeast strains AH109 (Clontech) were transformed with bait and prey cotransform constructs. Yeast diploids were selected on selection plates containing SD (Synthetic Dropout) medium lacking Leu, Trp, and His. The interactions between p53 and the SV40 large T-antigen (T), and between lamin (Lam) and the T served as positive and negative controls, respectively.

### *In Vivo* Co-immunoprecipitation Assays

*In vivo* co-immunoprecipitation (Co-IP) assays were carried out by transient protein expression in rice protoplasts. HA- tagged OsRRK1 and MYC- tagged LecRK constructs were coexpressed in rice protoplasts, extracted in the buffer [50 mM Tris-HCl pH 7.5, 150 mM NaCl, 10% glycerol, 0.1% NP-40, 1 mM PMSF, plant protease inhibitor cocktail (Roche)], immunoprecipitated with anti-MYC antibody, then detected by the anti-HA (MBL, Catalog: M180) and anti-Myc (MBL, Catalog: M047), respectively.

### Plasmid Constructs and Rice Transformation

To make an overexpression construct (OE-*OsRRK1*), a 1179-bp cDNA fragment encoding the full-length of *OsRRK1* was PCR-amplified from the cDNA library of Kasalath using a pair of primers, OE-*OsRRK1*-F and OE-*OsRRK1*-R (Supplementary Table S1). The amplified DNA fragments were ligated with pCXUN-vector, which was digested with *XcmI*. The pCXUN-vector contains the maize *ubiquitin* promoter and a nos terminator.

The *OsRRK1*-RNAi constructs were generated by an overlapping PCR approach (Chen et al., 2009). Briefly, in the first-round PCR, two 521-bp fragments between +569 and +1089 (relative to the ATG at +1 bp) were amplified from the cDNA library of 9311 using the primers RNAi-F and RNAi-R1, RNAi-F and RNAi-R2. The PDK intron loop sequence fragment was amplified from pHAN using the primers PDK-F and PDK-R. Primers RNAi-R1 and PDK-F were used to introduce complementary adapters to the amplified fragments, as RNAi-R2 and PDK-R. The three amplified fragments were fused together as an inverted-repeat cassette in the second-round PCR by using a single RNAi-F primer. The resulting fragment was then directly cloned into the plant expression pCXUN-vector. All the primers used for vector construction are shown in Supplementary Table S1.

The constructs were introduced into *Agrobacterium tumefaciens* EHA105 via electroporation. The *Agrobacterium*-mediated transformations of rice (Hejiang19) were carried out as previously described (Chen et al., 2007).

### Measurement of the Leaf Rolling Index and Leaf Erection Index

To determine the LRI, two measurements were taken, *L*_w_ (expand the leaf blade and determine the greatest width of the leaf blade) and *L*_n_ (measure the natural distance of the leaf blade margins at the same location on the leaf where *L*_w_ was measured). LRI was calculated as LRI (%) = (*L*_w_ -*L*_n_)/*L*_w_ × 100%.

To determine the LEI, two measurements were taken, *L*_nl_ (the linear distance between the lamina joint and the tip of the leaf blade in the natural position) and *L*_sl_ (the length of the straightened leaf). LEI was calculated as LEI (%) = *L*_nl_/*L*_sl_ × 100% (Shi et al., 2007).

Data were collected from the flag leaves of 20 individual plants at the heading stage.

### Southern Blot Analysis

A probe (Supplementary Table S1) was labeled with [α-^32^P] dCTP using the Prime-a-Gene labeling system (Promega). Twenty microgram genomic DNA was digested with *EcoR*I restriction enzyme (Fermentas), then separated on a 1% agarose gel and blotted onto Hybond-N^+^ nylon membrane (Amersham Biosciences). The membranes were prehybridized for 3 h at 65°C and the hybridization buffer was refreshed with the labeled probe. The membranes were then incubated for 12 h at 65°C. Washing was conducted at 65°C for 15 min in 2 × SSC and 0.2% SDS, and subsequently for 15 min at 65°C in 1 × SSC and 0.1% SDS. The membranes were then exposed to storage phosphor screens (Amersham Biosciences), and the hybridization signals were detected using a Typhoon PhosphorImager (Amersham Biosciences).

### Quantitative Real-time PCR (qRT-PCR)

Total RNA of various rice tissues including radicle and plumule in 48 h after emergence, root and leaf in the second tillering stage, flag leaf, second leaf from the top leaf, stem, leaf sheath, and young panicle at the heading stage were isolated using TRIzol reagent (TaKaRa) according to the manufacturer’s protocol. One microgram RNA was treated with DNase I (Fermentas) to remove genomic DNA and then used to synthesize cDNA with a RevertAid^TM^ First Strand cDNA Synthesis Kit (Fermentas) following the manufacturer’s recommendations. The cDNA was then amplified by specific primers and SYBR Green PCR Master Mix (Applied Biosystems) in a CFX96 Real-Time System (Bio-Rad). The analysis of the results was performed as previously described (Wei et al., 2009). All primers used in this study are listed in Supplementary Table S1.

### Histology and Microscopy Observation

To determine the detailed structure of leaf, mature flag leaves containing at least the bottom 1/2 of the tissues were used for a paraffin cross-section assay. Leaves were fixed in 70% formalin–acetic acid–alcohol solution (FAA) for 24 h at 4°C. After serial dehydration in various concentrations of ethanol, the samples were transferred into xylene and then embedded in paraffin. Sections (10 μm thick) were cut with a microtome (Leica RM2245) and mounted on microscope slides. Slices were spread on a platform at 40°C overnight and stained using 0.5% Toluidine Blue O at 37°C for 30 min. After dewaxing and rehydrating, the slices were examined and photographed using a LEICA CTR5000B microscope. Bulliform cell area was measured with Image J software^1^.

### Analysis of Alignments and Phylogenesis

The BLASTP program^2^ was used to identify the homologous sequence of *OsRRK1*. Before phylogenetic analysis, multiple sequence alignments were generated using ClustalX Version 1.83 (Thompson et al., 1997). A phylogenetic tree was constructed by MEGA Version 5.1.0 using the neighbor-joining (NJ) method. Bootstrap analyses of 1,000 replicates were carried out.

### BPH Host Choice Test

The host choice tests were conducted on 4-week-old rice plants in a cup (10 cm in diameter). Two WT plants and two OE-*OsRRK1* plants were planted at opposite ends of roughly perpendicular diagonals. Thirty-fourth-instar BPH nymphs were then placed in the middle of the cup and the number of nymphs that settled on each plant was recorded each day after infestation. The experiment was repeated twice, each time with 20 biological replicates.

### RNA-Sequencing and Data Analysis

The flag leaves of both Hejiang19 and OE-25 at the five-leaf stage were collected for RNA-sequencing analysis, with each sample containing a pool of 10 plants, each rice variety containing three biological replicates. Total RNAs were prepared using RNAiso Plus according to the manufacturer’s protocol (TaKaRa Code: D9108A). Half of RNA was performed to transcription expression analysis by Shanghai Biotechnology Corporation, and the rest was used to verify the RNA-sequencing results by qRT-PCR. All subsequent procedures, including mRNA purification, cDNA preparation, end repair of cDNA, adaptor ligation, and cDNA amplification were performed according to the manufacturer’s protocols accompanying the mRNA-Seq Sample Preparation Kit (Illumina). Each library had an insert size of 45 bp, and sequences of 50 bp on one end (1^∗^50 bp) were generated via Illumina HiSeq2000.

Cufflinks (version: 2.0.2^3^) was used to calculate the FPKM value of every transcript. The *P*-values of different expressions were calculated using Fisher’s exact test. We used *P* < 0.05 and the absolute value of log_2_FC ≥ 0.585 as the threshold to judge the significance of each gene expression difference (Trapnell et al., 2010). Cluster analysis was performed and heat maps generated with the Genesis software based on a hierarchy (version: 1.7.6^4^) (Sturn et al., 2002). For pathway analysis, we mapped all DEGs using the MapMan package with the Osa_MSU_v7 mapping file (Thimm et al., 2004).

### Accession Numbers

Sequence data for OsRRK1 can be found in the GenBank database under accession number KY347802. The RNA-Seq raw data were submitted to Short Read Archive at NCBI with accession numbers of SAMN06448985, SAMN06448986, SAMN06448987, SAMN06448988, SAMN06448989, and SAMN06448990.

## Results

### Characterization of the *OsRRK1* Gene

In the previous study, OsLecRK was used as a bait to screen a rice cDNA expression library established in a yeast two-hybrid system (Cheng et al., 2013). We isolated a full-length cDNA encoding a RLCK, which interacted with OsLecRK by a yeast two-hybrid assay (Supplementary Figure S1A) and co-immunoprecipitation (Supplementary Figure S1B). BLAST analysis in the NCBI database showed that this RLCK exhibits 57% amino acid sequence identity with AtRRK1 (Rop-interacting receptor-like kinase 1) from *Arabidopsis*, we named this gene *OsRRK1* (LOC_Os06g47820). The full-length cDNA of *OsRRK1* is 1567-bp long, and consists of a 237-bp 5’UTR, 1179-bp coding region and 151-bp 3’UTR (Supplementary Figure S2). The open reading frame (ORF) of *OsRRK1* comprises six exons and five introns. It is predicted that it encodes a polypeptide of 392 amino acids with a serine-threonine/tyrosine-protein kinase catalytic (STYKc) domain (Supplementary Figures S3A,B).

**FIGURE 1:**
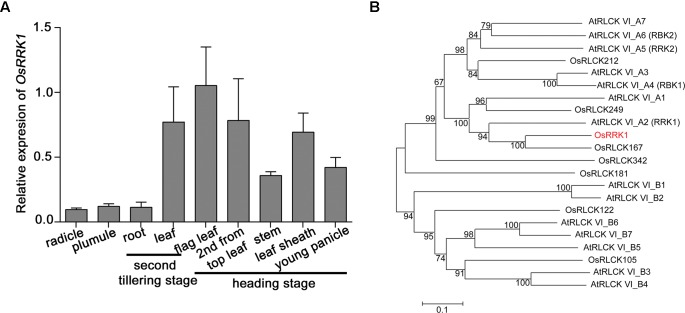
Characterization of the *OsRRK1* gene. **(A)** Expression of *OsRRK1* in various organs determined by qRT-PCR analysis. Rice *ACTIN1* gene was used as an internal control. Error bars represent the SD of transcript levels determined from three independent replicates. **(B)** Phylogenetic analysis of OsRRK1 and its homologous protein with other RLCK VI sub-family members in *Arabidopsis* and rice by MEGA6.0 constructed using the Neighbor-Joining method. The OsRRK1 protein is shown in red. The GenBank accession numbers of OsRRK1 and its homologous proteins are as follows: OsRRK1: XP_015642177.1; OsRLCK212: XP_015642870.1; OsRLCK249: XP_015648938.1; OsRLCK167: XP_015634276.1; OsRLCK342: ABA95043.1; OsRLCK181: XP_015640273.1; OsRLCK122: XP_015632245.1; OsRLCK105: XP_015630796.1; AtRLCK VI_A1: NP_001078762.1; AtRLCK VI_A2: NP_179479.1; AtRLCK VI_A3: NP_201356.2; AtRLCK VI_A4: NP_568231.1; AtRLCK VI_A5: NP_198445.1; AtRLCK VI_A6: NP_001327889.1; AtRLCK VI_A7: NP_197392.2; AtRLCK VI_B1: NP_198595.1; AtRLCK VI_B2: NP_001321968.1; AtRLCK VI_B3: NP_001190918.1; AtRLCK VI_B4: NP_179266.2; AtRLCK VI_B5: NP_201199.1; AtRLCK VI_B6: NP_001319394.1; AtRLCK VI_B7: NP_173578.2.

The expression level of *OsRRK1* in various rice organs and at different development stages was determined by quantitative real-time PCR (qRT-PCR). This demonstrated that the expression level of *OsRRK1* was highest in leaves, especially in flag leaves, and then decreased gradually in leaf sheathes, young panicles, stems, plumules, radicles, and roots (**Figure 1A**).

*OsRRK1* is OsRLCK216 and belongs to the RLCK VI subfamily (Vij et al., 2008; Gao and Xue, 2012). In order to analyze the genetic relationship between *OsRRK1* and other RLCKs in rice and *Arabidopsis*, we constructed an unrooted phylogenetic tree for OsRRK1 and found that OsRRK1 exhibits relatively higher homology with OsRLCK167 and AtRLCK VI_A2 (AtRRK1) (Dorjgotov et al., 2009) (**Figure 1B**).

### Overexpression of *OsRRK1* Results in a Leaf Rolling Phenotype

In order to elucidate the function of *OsRRK1* in rice, an *OsRRK1* overexpression construct driven by the ubiquitin promoter was produced and introduced into WT plants via *A. tumefaciens*-mediated transformation. Seventeen positive transgenic lines (OE-*OsRRK1*) were generated and confirmed by Southern blot analysis (Supplementary Figure S4). Eight of them (OE-5, 7, 16, 19, 21, 22, 24, 25) had a single copy of *OsRRK1* and the others had multiple copies. We chose three single copy lines (OE-22, 24, and 25) for further analysis.

**FIGURE 2:**
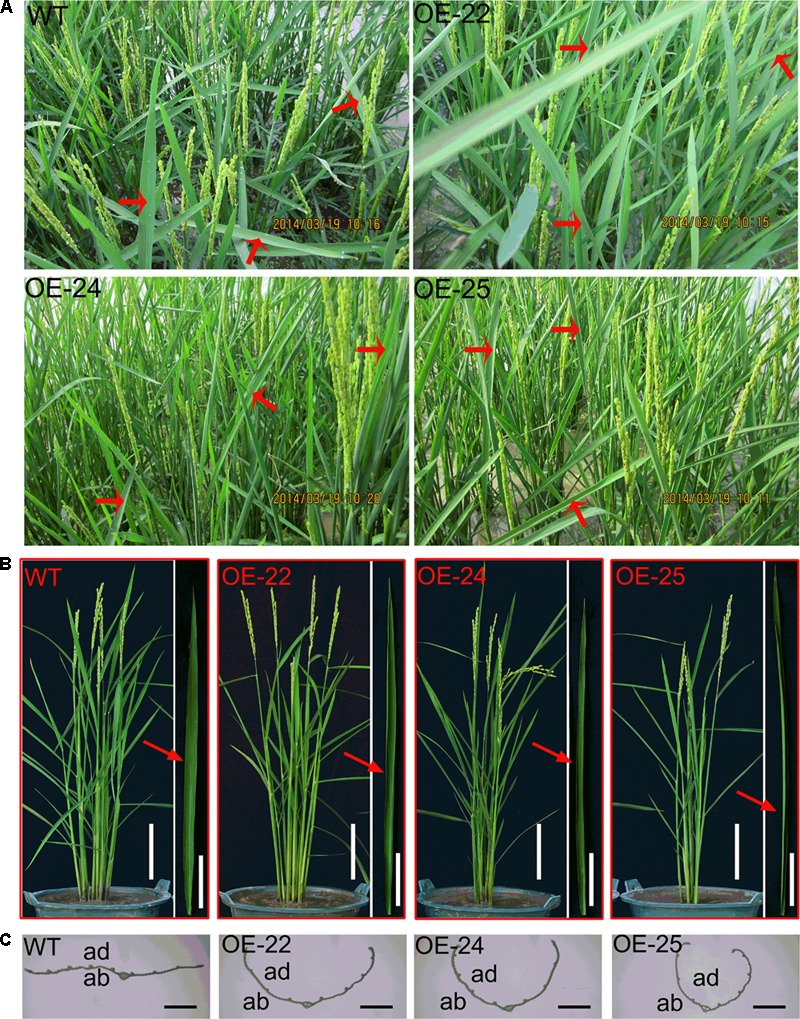
The leaf phenotype of the WT and OE-*OsRKK1* plants. **(A)** Morphology of WT plants and the OE-*OsRRK1* plants at the heading stage in the paddy field. Red arrow indicated the leaf morphology. Leaves of WT plants were flat, whereas the flag leaves of OE-*OsRRK1* plants were rolled to different degrees in the field. **(B)** Mature leaves of the WT and OE-*OsRRK1* plants at the heading stage in a bucket (Bar = 10 cm). The corresponding close-up images of the flag leaf architecture details are shown besides each bucket image (Bar = 5 cm). **(C)** Cross-section of the middle part of the flag leaf in the WT and the OE-*OsRRK1* plants at the heading stage. ad, adaxial; ab, abaxial. Bars = 2 mm.

Compared to WT plants, all of the OE-*OsRRK1* plants displayed the leaf rolling phenotype. During the heading stage, we were able to observe that leaves of WT plants were flat, whereas the flag leaves of OE-*OsRRK1* plants were rolled to different degrees in the field (**Figure 2A**) and the laboratory (**Figure 2B**). We transected the middle part of these leaves and found that the cross-section revealed rolling to varying degrees (**Figure 2C**).

**FIGURE 3:**
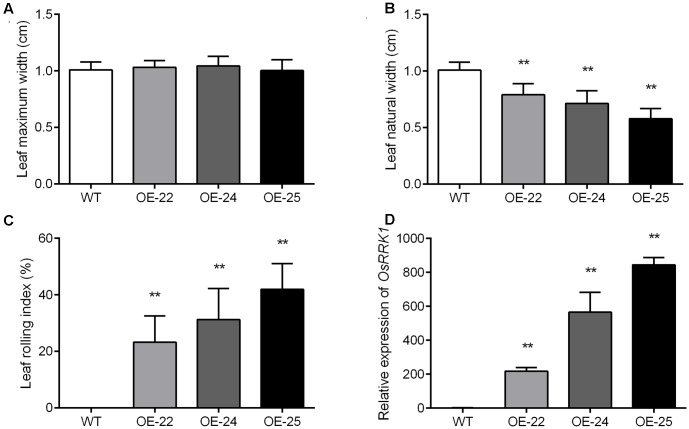
Evaluation of the degree of leaf rolling in WT and OE-*OsRRK1* plants. **(A,B)** Leaf maximum width **(A)** and leaf natural width **(B)** of the WT and OE-*OsRRK1* plants. Twenty plants of each line were measured at the heading stage. **(C)** LRI of the flag leaves of WT and the OE-*OsRRK1* plants based on the leaf maximum and natural width. Error bars indicate SD (*n* = 20). **(D)** The relative expression levels of *OsRRK1* in the WT and OE-*OsRRK1* plants. Flag leaves at the heading stage were used in this experiment. Values are the means ± SD of three biological replicates. ^∗^Student’s *t*-test, *P* < 0.05; ^∗∗^Student’s *t*-test, *P* < 0.01.

To evaluate the degree of leaf rolling accurately, we calculated the LRI, for which a higher value indicates a higher rolling degree (Shi et al., 2007). At the heading stage, we measured the maximum leaf width (*L*_w_) and natural leaf width (*L*_n_). The maximum width of flag leaves was not significantly different in WT plants and the OE-*OsRRK1* plants (**Figure 3A**). However, the natural width of flag leaves in OE-*OsRRK1* plants was significantly reduced compared to WT (**Figure 3B**). In WT plants, the natural width of flag leaves was almost the same as the maximum width, while in OE-*OsRRK1* plants the natural width of flag leaves was much smaller than the maximum width. The LRI of the WT plants was 0, while the LRIs of the OE-*OsRRK1* plants were at least 20% higher than that of the WT plants (**Figure 3C**). These results indicate that the leaf rolling of the OE-*OsRRK1* plants could be attributed to changes in the natural width of the leaf rather than the maximum width.

In general, leaf rolling leads to changes in the erectness of leaves (Shi et al., 2007). Thus, we measured the LEI, which quantifies how upright the leaves are. At the heading stage, we measured the linear distance between the lamina joint and the tip of the leaf blade (*L*_nl_) and the length of the straightened leaf in its natural position (*L*_sl_). The LEIs in the flag leaves of OE-*OsRRK1* plants were clearly different from those of the WT plants. The LEI calculations yielded values for the flag leaves of OE-*OsRRK1* plants all close to 100%, while the LEIs of the WT plants averaged 95.1 ± 3.08% (Supplementary Figure S5).

To explain the different degrees of rolling in the three OE-*OsRRK1* plants, we tested the expression level of *OsRRK1* in these transgenic plants by qRT-PCR analyses. The results demonstrated that the expression of *OsRRK1* in these transformants was upregulated to varying degrees. They were 200 times, 500 times, and 800 times upregulated in OE-22, OE-24, and OE-25, respectively (**Figure 3D**). The results revealed that with the increase of the *OsRRK1* relative expression level, the natural width of the flag leaves becomes smaller, so they may be negatively correlated. While with the increase of the *OsRRK1* relative expression level, the LRI also increase, so they may be positively correlated. However, LEI did not show significant correlation with the relative expression of *OsRRK1.*

To investigate the role of *OsRRK1* in rice leaf development further, RNAi analysis was also conducted. Unexpectedly, although the expression of *OsRRK1* was notably downregulated, no obvious change in leaf development was detected (Supplementary Figures S6A–C). The LRIs of all the RNAi plants were 0 in the flag leaves, which is similar to the WT plants. In addition, the LEIs of all the RNAi plants exhibited no obvious difference from the WT plants (Supplementary Figure S6D). *OsRLCK167 is* the closest homolog of *OsRRK1*. We compared the expression abundance of *OsRRK1* and *OsRLCK167* in WT rice leaves and found they showed a similar expression level in flag leaf at heading stage (Supplementary Figure S7A). And we found that the expression of *OsRLCK167* in RNAi plants showed no difference from the WT plants (Supplementary Figure S7B). Therefore, we suggested that functional redundancy might explain no changes in phenotypes by RNAi approaches. In addition, the transcript level of the RNAi plants was not inhibited completely, which was very possible these RNAi lines still have more than sufficient expression of OsRRK1 for normal function. Thus, we concluded that overexpression of *OsRRK1* results in a rolled-leaf phenotype, while moderate downregulation of *OsRRK1* was associated with no abnormality in leaf development.

### Bulliform Cell Number and Size Are Decreased in OE-*OsRRK1* Plants

**FIGURE 4:**
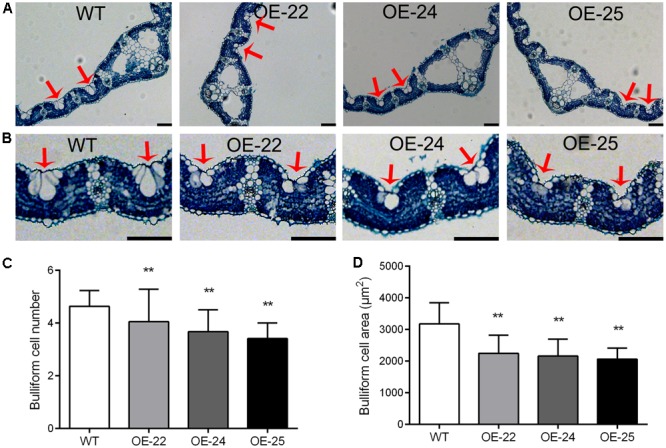
Bulliform cell number and size in the leaves of OE-*OsRRK1* plants. **(A)** Cross sections of flag leaves in WT and OE-*OsRRK1* plants. **(B)** Enlarged view of cell structure of flag leaves in WT and OE-*OsRRK1* plants. Bulliform cells are indicated by *red arrows*. Bars = 100 μm. **(C,D)** The bulliform cell number **(C)** and bulliform cell area **(D)** in the leaves of WT and OE-*OsRRK1* plants. Ten samples were investigated. ^∗^Student’s *t*-test, *P* < 0.05; ^∗∗^Student’s *t*-test, *P* < 0.01.

Bulliform cells are located between the vascular veins of the leaf in rice. Previous studies have demonstrated that changes in the number and area of the bulliform cells result in leaf rolling (Zhang et al., 2009, 2015; Li et al., 2010). Therefore, we performed transverse sectioning so that we could examine the leaves of OE-*OsRRK1* and WT plants. The bulliform cells were smaller and fewer in OE-*OsRRK1* leaves than in the WT leaves, whereas no significant changes were found for other cell types and their arrangements between OE-*OsRRK1* and the WT leaves (**Figures 4A,B**). The bulliform cells of WT plants were typically arranged in groups of 4.64 ± 0.59 cells, whereas the bulliform cells in OE-*OsRRK1* leaves were only in groups of 4.05 ± 1.23, 3.68 ± 0.83, and 3.41 ± 0.59 cells in OE-22, OE-24, and OE-25, respectively (**Figure 4C**). In addition, the bulliform cell area in OE-*OsRRK1* leaves was reduced compared to WT leaves (**Figure 4D**). We also found that with the increase of the *OsRRK1* expression level, the number of bulliform cells and the average cell area were all decreased, suggesting they may be negatively correlated. These results suggest that overexpression of *OsRRK1* decreased bulliform cell number and size, which resulted in leaf rolling.

### Overexpression of *OsRRK1* in Rice Conferred an Antixenosis Effect to BPH

**FIGURE 5:**
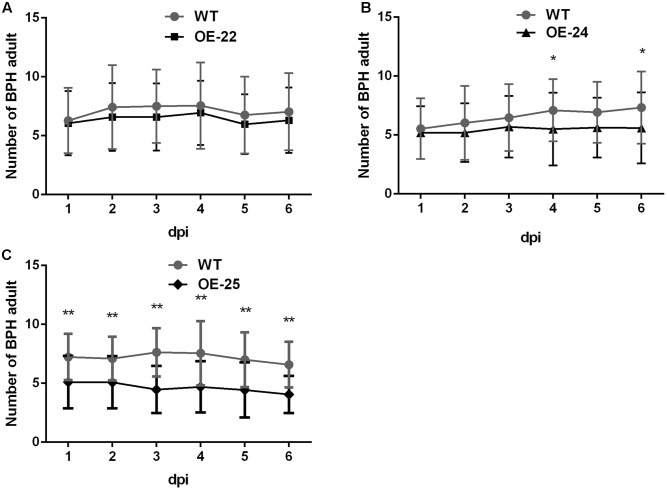
Two-host choice test for BPHs on WT and OE-*OsRRK1* plants. **(A)** No significant difference between BPHs settling on WT plants and OE-22 plants. **(B)** Significant difference between BPHs settling on WT plants and OE-24 plants was only observed on the fourth day and the sixth day. **(C)** BPHs congregated on the WT plants to a greater extent than on OE-25 plants and there was a significant difference from the first day to the sixth day. ^∗^Student’s *t*-test, *P* < 0.05; ^∗∗^Student’s *t*-test, *P* < 0.01.

OsRRK1 was identified as an interactor of OsLecRK, which was involved in defense to BPH (Cheng et al., 2013). We compared the performance of BPH on OE*-OsRRK1* and WT plants to determine whether *OsRRK1* is involved in defense to this herbivorous insect of rice. Host preference was assessed on the basis of the number of BPHs that settled on WT and OE*-OsRRK1* plants after releasing BPHs into the center of containers where BPHs could choose between WT and OE*-OsRRK1* plants. Comparing the OE*-*22 and WT plants, there was no significant difference in the number of BPHs on each from the first day to the sixth day (**Figure 5A**). However, more insects choose to settle on the WT plants than on the OE-24 and OE-25 plants. From the fourth day, the number of BPHs settled on each plant type was significantly different between the OE-24 and WT plants (**Figure 5B**). For the OE*-*25 and WT plants, a significant difference of BPH choice was observed from the first day to the sixth day (**Figure 5C**). These results indicated that *OsRRK1* confers defense to BPH attacks via antixenosis, which is known to be an important mechanism of plant defense against insect attacks (Walling and Thompson, 2012). As the *OsRRK1* overexpression level can be ranked OE*-*25 > OE*-*24 > OE*-*22, our results suggest that the level of defense is determined by the accumulation level of *OsRRK1* transcripts.

### Pleiotropic Roles of *OsRRK1* in Multiple Plant Developmental Processes

**FIGURE 6:**
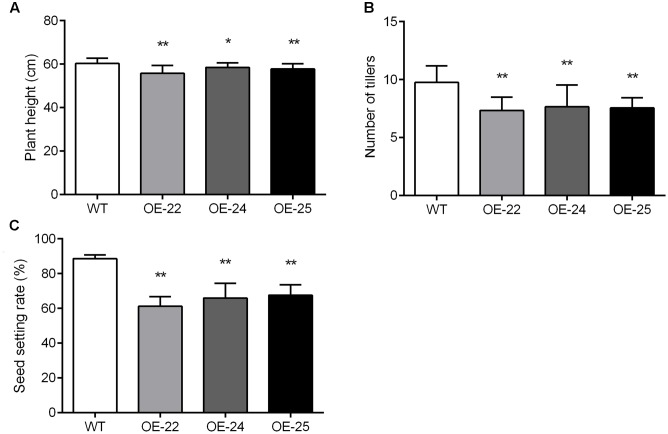
Pleiotropic effects of the *OsRRK1* gene. **(A,B)** Plant height **(A)** and tiller number **(B)** in WT plants and OE-*OsRRK1* plants at the heading stage. Data are averages for 20 plants. **(C)** The seed setting rate of WT plants and OE-*OsRRK1* plants. Seeds from 20 plants were measured after harvest. The seed setting rate was determined as: (filled grain number per panicle)/(grain number per panicle)^∗^100%. Asterisks indicate a significant difference between WT plants and OE-OsRRK1 plants according to a *t*-test, ^∗^Student’s *t*-test, *P* < 0.05; ^∗∗^Student’s *t*-test, *P* < 0.01.

In addition to rolled and erect leaves, other phenotype changes in OE-*OsRRK1* plants were also observed. Compared with WT plants, the plant height, tiller numbers and seed setting rate of OE*-OsRRK1* plants were significantly reduced. The height of WT plants was 60.28 ± 2.45 cm, whereas the height was 55.77 ± 3.65 cm, 58.51 ± 2.15 cm, and 57.79 ± 2.38 cm in OE-22, OE-24, and OE-25, respectively (**Figure 6A**). There were about 9.75 ± 1.45 tillers in WT plants, but only 7.35 ± 1.14, 7.65 ± 1.90, and 7.55 ± 0.89 tillers in OE-22, OE-24, and OE-25, respectively (**Figure 6B**). In WT plants, the seed setting rate was about 88.61% ± 2.09%, but was 61.2% ± 5.48%, 65.96% ± 8.38%, and 67.67% ± 6% in OE-22, OE-24, and OE-25, respectively (**Figure 6C**). These results indicate that overexpression of *OsRRK1* changed multiple plant developmental processes.

### Transcript Profiles Are Distinct in WT Plants and OE-*OsRRK1* Plants

To understand the molecular mechanism of rice development and defense to BPH mediated by *OsRRK1*, the expression profiles of WT plants Hejiang19 (H) and a line of OE*-OsRRK1* plants (OE-25, abbreviated as OE hereafter) were determined using deep RNA-sequencing. As a result, 116.4 million paired-end sequence reads of 50-bp in length were generated from the six samples. After removing low-quality reads, a total of 113.8 million high quality clean reads were retained, of which more than 97% were aligned to the reference genome using TopHat (Supplementary Table S2).

One fundamental use of transcriptome sequencing is the analysis of differentially expressed genes (DEGs) between groups (Wang et al., 2009). In our study, we defined DEGs as the transcripts showing at least a 1.5-fold change in the FPKM (fragments per kilobase of exon per million fragments mapped) (log_2_FC ≥ 0.585 or log_2_FC ≤ –0.585) and a *P*-value < 0.05. In total, 625 DEGs were detected among the paired comparisons (H vs. OE), including 366 upregulated and 259 downregulated genes. DEGs in H and OE of the three biological replicates were hierarchically clustered in the heat map (Supplementary Figure S8). The majority of DEGs had similar expression patterns among three biological replicates, showing consistent upregulation or downregulation.

**FIGURE 7:**
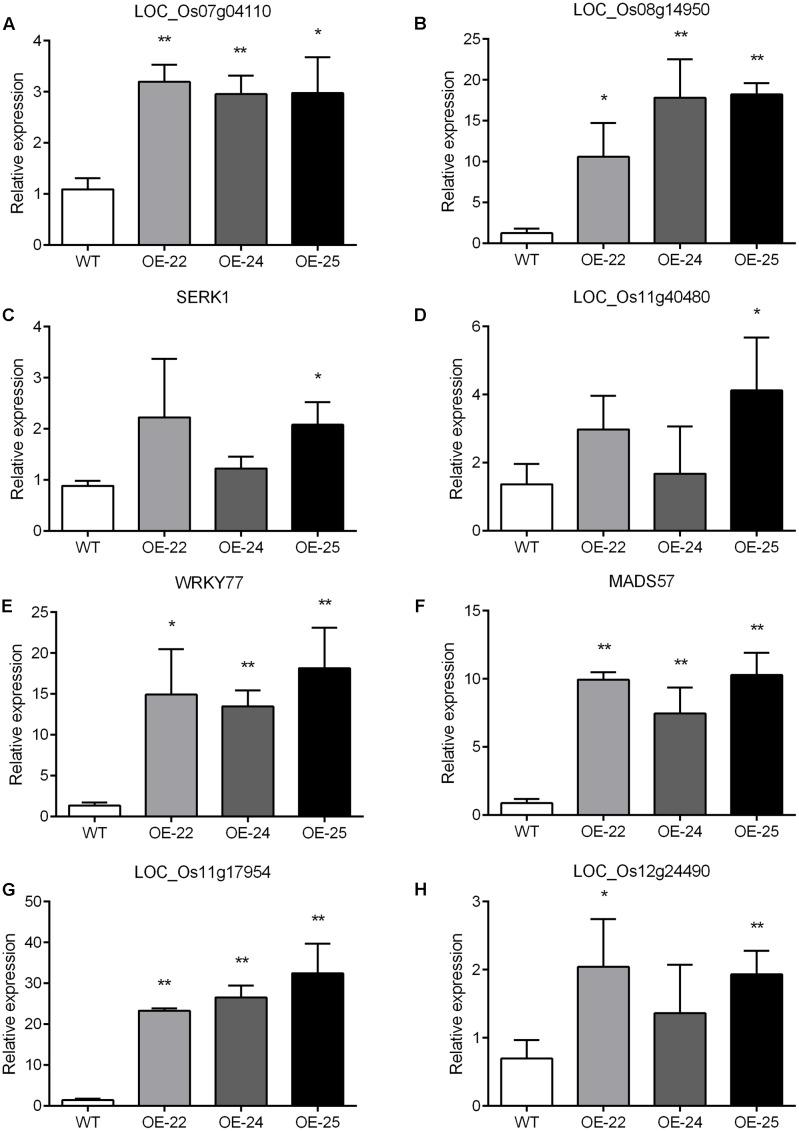
**(A–H)** qRT-PCR analysis of differentially expressed genes detected by the transcriptomic analysis. SERK1: LOC_Os11g31540; WRKY77: LOC_Os01g40260; MADS57: LOC_Os02g49840. Significant differences are indicated by ^∗^*P* < 0.05, ^∗∗^*P* < 0.01 (Student’s *t*-test).

To enhance our understanding of the biological function of DEGs, those assigned to MapMan pathways and important classifications are provided in **Table 1**. Receptor kinases and receptor-like cytoplasmatic kinase VII-related genes are important components of signal transduction and play roles in plant development. In this study, 43 genes encoding receptor kinases and receptor-like cytoplasmatic kinase VII were upregulated, 11 genes encoding receptor kinases were downregulated (**Table 1**). Among them, legume lectins beta domain containing protein (LOC_Os07g04110) and receptor-like protein kinase 2 precursor (LOC_Os08g14950) are involved in plant resistance; brassinosteroid insensitive 1-associated receptor kinase 1 (LOC_Os11g31540) and receptor-like protein kinase 2 (LOC_Os11g40480) are involved in plant development. To verify the RNA-sequencing results, the expressions of the aforementioned four genes were analyzed by qRT-PCR with gene specific primers. The qRT-PCR results were consistent with RNA-sequencing data (Supplementary Table S3). Compared to WT, all of them were upregulated in all three OE-*OsRRK1* plants (**Figures 7A–D**).

**Table 1:** Pathway classification by MapMan.

Pathways	H_OE	
	U	D
Receptor kinases and receptor-like cytoplasmic kinases	43	11
Transcription factors	17	14
Biotic stress	22	12
Development	13	10
Hormone	7	6
Abiotic stress	4	3
Short chain dehydrogenase/reductase (SDR)	5	1
Light signaling	4	1
ABC transporters and multidrug resistance systems	3	0
Ca^2+^ signaling	2	1

*H_OE, total DEG number in comparisons of Hejiang19 and OE-25. U, upregulated;D, downregulated.*

Transcription factors (TFs) also play important roles in defense responses and plant development (Wang et al., 2012). There are 31 TF-related DEGs between the WT and OE plants, including 17 upregulated genes and 14 downregulated genes (**Table 1**). In previous studies, overexpression of *OsWRKY77* (LOC_Os01g40260) repressed growth of a pathogen by enhancing expression of defense-related *PR1*, *PR2*, and *PR5* genes, and overexpression of *OsMADS57* (LOC_Os02g49840) resulted in increased tillers (Wang et al., 2008; Guo et al., 2013; Lan et al., 2013). Moreover, MYB domain TFs and ZF domain TFs also participate in plant development and resistance (Sugimoto et al., 2000; Shin et al., 2002; Wang et al., 2008). We also confirmed these four TF gene expressions in WT and OE plants by qRT-PCR. The qRT-PCR results showed that all of them were consistent with the transcriptome data (Supplementary Table S3) and were upregulated in all three OE-*OsRRK1* plants (**Figures 7E–H**). *OsRRK1* may operate by means of transducing signals activated by receptor kinases and then regulating related TFs, finally resulting in leaf rolling and a BPH-defense phenotype.

In rice, at least 35 leaf rolling mutants have been reported, 13 genes of which were related to the bulliform cells; these include *RL14, ACL1, SRL1* and *SLL1*. However, none of these genes were found differentially expressed between WT and OE-25 plants in the transcriptome data (Supplementary Table S3). These results suggest that overexpression of the *OsRRK1* gene resulting in leaf rolling is independent of the previously reported leaf rolling-related genes.

## Discussion

The RLCKs represent a large gene family in plants with diverse biological roles, which include development and stress responses (Vij et al., 2008; Liu et al., 2016). They are divided into 13 subfamilies in *Arabidopsis* and 17 subfamilies in rice based on phylogenetic clades of amino acid sequences (Shiu et al., 2004). Most well studied *RLCK* genes belong to the RLCK VII subfamily, while there have been few studies of the RLCK VI subfamily. In *Arabidopsis*, only four RLCK VI subfamily genes have been studied. Rop binding protein kinases 1 and 2 (RBK1 and 2) directly bind to AtRop4 GTPase, which has emerged as a central regulator of diverse signaling pathways in plant growth and pathogen defense (Molendijk et al., 2008). Rop-interacting receptor-like kinase 1 and 2 (AtRRK1 and 2) kinases could be specifically activated by GTP-bound Rop GTPases *in vitro* (Dorjgotov et al., 2009). However, the characterization of RLCK VI subfamily genes in rice has not been reported. In this study, we identified a RLCK VI subfamily gene *OsRRK1* as a potential novel molecular interactor of *OsLecRK* (Supplementary Figure S7). The overexpression of *OsRRK1* caused leaf rolling, agriculturally relevant traits and defense to BPH in rice, indicating that the RLCK VI subfamily also may play roles in both rice development and immunity.

**FIGURE 8:**
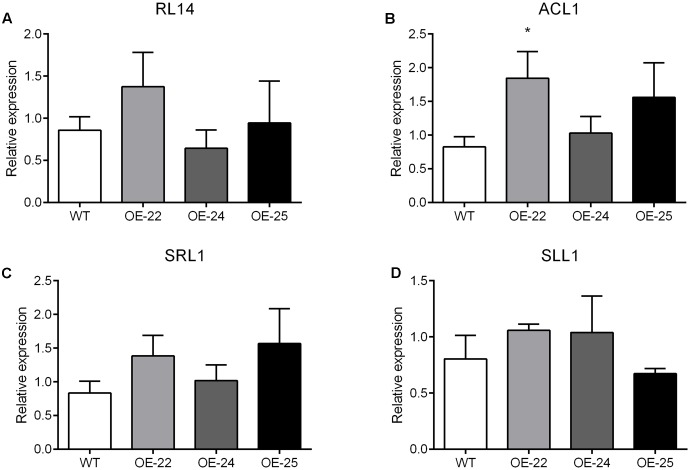
**(A–D)** qRT-PCR analysis of the reported genes related to leaf rolling. Significant differences are indicated by ^∗^*P* < 0.05, (Student’s *t*-test).

To date, many studies have been performed to characterize genes controlling leaf rolling by analysis of mutants in rice. More than 35 leaf rolling mutants have been reported, of which 13 genes have been cloned and affected leaf rolling through the regulation of bulliform cells. For example, RL14 encodes a 2OG-Fe (II) oxygenase. The *rl14* mutant reduced bulliform cell size and caused inward leaf rolling (Fang et al., 2012). ACL1, encoding a protein with unknown conserved functional domains, positively regulated bulliform cell development since the enhanced expression of ACL1 increased bulliform cell number and resulted in outward leaf rolling (Li et al., 2010). SRL1, encoding a putative glycosylphosphatidylinositol-anchored protein, negatively regulated the bulliform cell number since its loss-of-function mutant increased bulliform cell number and led to inward leaf rolling (Xiang et al., 2012). SLL1 encodes a transcriptional factor of the MYB family. The *sll1* mutant also displays inward leaf rolling due to defective development of abaxial sclerenchymatous cells and formation of bulliform cells on the abaxial epidermis (Zhang et al., 2009). In this study, *OsRRK1* negatively regulates the bulliform cell number and size. The degree of rolling was positively correlated with the expression level of *OsRRK1*. Through analysis of transcriptome and qRT-PCR data between the WT and OE-25 plants, none of the 13 reported genes associated with leaf rolling were found differentially expressed (Supplementary Table S3 and **Figures 8A–D**). These results suggest that leaf rolling resulting from overexpression of *OsRRK1* was activated by a novel pathway, which was independent of these previously reported genes related to leaf rolling.

*OsRRK1* encodes an intracellular protein with a STYKc domain (Supplementary Figure S3) and interacts with OsLecRK to confer defense to BPH attacks via antixenosis (**Figures 5B,C**), which is known to be one of the major mechanisms of plant defense against insect attacks (Cheng et al., 2013). Previous studies have provided evidence that RLCKs can be phosphorylated by receptor kinases. For example, OsCERK1 (chitin elicitor receptor kinase 1), a plasma membrane protein, phosphorylated OsRLCK185 with a STKc domain. OsRLCK185 is a possible transmitter which links the PRR OsCERK1 with MAP kinase cascades and regulates chitin induced immune responses (Yamaguchi et al., 2013). Other RLCKs, such as OsRLCK57, OsRLCK107, and OsRLCK176 with the STYKc domain, interact with OsBRI1, a rice brassinosteroid receptor, and they also regulate immune responses by the immune receptor XA21 (Zhou et al., 2016). OsRRK1 is an interactor of OsLecRK. The BPH resistance protein BPH3 is also composed of three lectin receptor kinases (OsLeckRK1-3). Both of these lectin receptor kinases confer rice resistance to BPH. Thus, we hypothesize that OsRRK1 may be similar to other RLCKs. It can be phosphorylated by OsLecRK, which could recognize HAMPs (herbivore-associated molecular patterns), and subsequently trigger immune responses to BPH. However, how OsRRK1 accepts signals from OsLecRK and what proteins are activated by OsRRK1 remain to be determined.

To understand the molecular mechanism of rice development and defense to BPH mediated by *OsRRK1*, we determined the expression profiles of WT plants and OE*-OsRRK1* plants by deep RNA sequencing. We found that many DEGs were involved in receptor kinases and transcription factors. Receptor kinases are pivotal components of signal transduction and play roles in plant development. In this study, we selected four genes, LOC_Os07g04110, LOC_Os08g14950, LOC_Os11g31540, and LOC_Os11g40480 for qRT-PCR confirmation (**Figures 7A–D**). In rice, the functions of these four genes are unknown. By analysis of homologous genes in *Arabidopsis*, we found that they act roles either in the process of plant development or plant defense. Perception of pathogen (or microbe)-associated molecular patterns (PAMPs/MAMPs) by pattern recognition receptors (PRRs) is a key component of plant innate immunity. The *Arabidopsis* EF-Tu receptor (EFR) recognizes the bacterial elongation factor Tu (EF-Tu) and confers broad-spectrum bacterial disease resistance (Schoonbeek et al., 2015). SERK1 is essential to the early events of BR signaling, which can regulate plant growth and development (Gou et al., 2012). Besides *OsLecRK*, *OsRRK1* may interact with other receptor kinases like the aforementioned ones to influence rice growth and development.

Transcription factors also play key roles in defense response and plant development (Wang et al., 2012). In this study, we also selected four genes, LOC_Os01g40260, LOC_Os02g49840, LOC_Os11g17954, and LOC_Os12g24490 for qRT-PCR analysis (**Figures 7E–H**). LOC_Os01g40260 (*OsWRKY77*) is a positive regulator of PR gene expression and basal resistance to the bacterial pathogen PstDC3000 (Lan et al., 2013). In *OsRRK1* overexpression lines, the expression of *OsWRKY77* was upregulated that could improve defense to BPH attacks. Overexpression of LOC_Os02g49840 (*OsMADS57*) can increase rice tillers by interacting with other proteins (Guo et al., 2013), and it was upregulated in *OsRRK1* overexpression lines. However, *OsRRK1* overexpression lines had reduced number of tillers compared to WT plants. We thought that there are a number of factors that affect the numbers of tillers, *OsMADS57* maybe just one of them. Even though the expression of *OsMADS57* was upregulated, some other genes may also affect the numbers of tillers in *OsRRK1* overexpression lines. LOC_Os11g17954 is a MYB domain TFs. LOC_Os12g24490 is a ZF domain TFs. Both MYB and ZF domain TFs participate in plant development and defense. For example, *AtMYB21* confers some features of constitutive photomorphogenesis even in the dark, and causes seedling lethality (Shin et al., 2002). NtMYB2 is involved in the stress response of the retrotransposon and defense-related genes (Sugimoto et al., 2000). Many members of the ZF subfamily are regulated by abiotic or biotic stresses, suggesting that they could have an effective role in stress tolerance (Wang et al., 2008). OsRRK1 could activate these TF genes to control rice leaf rolling and defense to BPH.

In addition to receptor kinases and TFs, 34 DEGs were involved in biotic stress (**Table 1**). Among them, 16 genes encode PR-proteins, which include NBS-LRR disease resistance protein. Plant NBS-LRR disease resistance protein is the largest protein family involved in disease resistance, pathogen sensing and host defense (Moffett et al., 2002; DeYoung and Innes, 2006). Previous studies have shown that BPH resistance genes can activate PR-proteins (Du et al., 2009). OsRRK1 conferred antixenosis to BPH and may also be related to the PR-protein. In addition, 33 DEGs were involved in development (**Table 1**). Although their functions in rice remain to be elucidated, the expression changes still reflect their role in OE-*OsRRK1* development. Thirteen DEGs are related to hormones (**Table 1**). Hormone signaling pathways play pivotal roles in plant development and defense. We found traditional defense hormones-related genes including three JA-related genes and two SA-related DEGs. It has been reported that JA and SA are related to defense against BPH (Du et al., 2009; Zhao et al., 2016). Three auxin-related genes were also found amongst the DEGs. Auxin biosynthesis affects the development of plants.

In summary, we characterized a gene that regulates not only development but also plant immunity. Moreover, the phenotypes of leaf rolling and BPH defense were positively correlated to the expression level of *OsRRK1.* This feature has the potential to facilitate artificial control of these phenotypes and may eventually contribute to the development of desired rice variety.

## Author Contributions

YM, BD, and GH designed the research; YM, YZh, XS, SS, YZe, YW, RC, AY, and LZ performed research; YM, BD, and GH analyzed data and wrote the manuscript. All authors read and approved the manuscript.

## Conflict of Interest Statement

The authors declare that the research was conducted in the absence of any commercial or financial relationships that could be construed as a potential conflict of interest.

## Footnotes

1 http://rsbweb.nih.gov/ij/

2 https://blast.ncbi.nlm.nih.gov

3 http://cufflinks.cbcb.umd.edu

4 http://genome.tugraz.at

## Abbreviations

BPH: brown planthopper
LEI: leaf erect index
LRI: leaf rolling index
OE: overexpression
RNAi: RNA interference

## References

[B1] Ao Y. Li Z. Feng D. Xiong F. Liu J. Li J. F. (2014). OsCERK1 and OsRLCK176 play important roles in peptidoglycan and chitin signaling in rice innate immunity. *Plant J.* 80 1072–1084. 10.1111/tpj.12710 25335639

[B2] Bowman J. L. Eshed Y. Baum S. F. (2002). Establishment of polarity in angiosperm lateral organs. *Trends Genet.* 18 134–141. 10.1016/S0168-9525(01)02601-4 11858837

[B3] Chen R. Zhao X. Shao Z. Wei Z. Wang Y. Zhu L. (2007). Rice UDP-glucose pyrophosphorylase1 is essential for pollen callose deposition and its cosuppression results in a new type of thermosensitive genic male sterility. *Plant Cell* 19 847–861. 10.1105/tpc.106.044123 17400897 PMC1867369

[B4] Chen S. Songkumarn P. Liu J. Wang G. L. (2009). A versatile zero background T-vector system for gene cloning and functional genomics. *Plant Physiol.* 150 1111–1121. 10.1104/pp.109.137125 19403729 PMC2705043

[B5] Cheng X. Wu Y. Guo J. Du B. Chen R. Zhu L. (2013). A rice lectin receptor-like kinase that is involved in innate immune responses also contributes to seed germination. *Plant J.* 76 687–698. 10.1111/tpj.12328 24033867 PMC4285754

[B6] DeYoung B. J. Innes R. W. (2006). Plant NBS-LRR proteins in pathogen sensing and host defense. *Nat. Immunol.* 7 1243–1249. 10.1038/ni1410 17110940 PMC1973153

[B7] Dorjgotov D. Jurca M. E. Fodor-Dunai C. Szucs A. Otvos K. Klement E. (2009). Plant Rho-type (Rop) GTPase-dependent activation of receptor-like cytoplasmic kinases in vitro. *FEBS Lett.* 583 1175–1182. 10.1016/j.febslet.2009.02.047 19285078

[B8] Du B. Zhang W. Liu B. Hu J. Wei Z. Shi Z. (2009). Identification and characterization of *Bph14*, a gene conferring resistance to brown planthopper in rice. *Proc. Natl. Acad. Sci. U.S.A.* 106 22163–22168. 10.1073/pnas.0912139106 20018701 PMC2793316

[B9] Dubouzet J. G. Maeda S. Sugano S. Ohtake M. Hayashi N. Ichikawa T. (2011). Screening for resistance against *Pseudomonas syringae* in rice-FOX *Arabidopsis* lines identified a putative receptor-like cytoplasmic kinase gene that confers resistance to major bacterial and fungal pathogens in *Arabidopsis* and rice. *Plant Biotechnol. J.* 9 466–485. 10.1111/j.1467-7652.2010.00568.x 20955180 PMC3118280

[B10] Fang L. Zhao F. Cong Y. Sang X. Du Q. Wang D. (2012). Rolling-leaf14 is a 2OG-Fe (II) oxygenase family protein that modulates rice leaf rolling by affecting secondary cell wall formation in leaves. *Plant Biotechnol. J.* 10 524–532. 10.1111/j.1467-7652.2012.00679.x 22329407

[B11] Gao L. Xue H. (2012). Global analysis of expression profiles of rice receptor-like kinase genes. *Mol. Plant* 5 143–153. 10.1093/mp/ssr062 21765177

[B12] Gou X. Yin H. He K. Du J. Yi J. Xu S. (2012). Genetic evidence for an indispensable role of somatic embryogenesis receptor kinases in brassinosteroid signaling. *PLOS Genet.* 8:e1002452. 10.1371/journal.pgen.1002452 22253607 PMC3257278

[B13] Guo S. Xu Y. Liu H. Mao Z. Zhang C. Ma Y. (2013). The interaction between OsMADS57 and OsTB1 modulates rice tillering via DWARF14. *Nat. Commun.* 4 1566. 10.1038/ncomms2542 23463009 PMC3615354

[B14] Itoh J. Nonomura K. Ikeda K. Yamaki S. Inukai Y. Yamagishi H. (2005). Rice plant development: from zygote to spikelet. *Plant Cell Physiol.* 46 23–47. 10.1093/pcp/pci501 15659435

[B15] Jane W. N. Chiang S. H. T. (1991). Morphology and development of bulliform cells in *Arundo formosana* Hack. *Taiwania Int. J. Life Sci.* 36 85–96.

[B16] Lan A. Huang J. Zhao W. Peng Y. Chen Z. Kang D. (2013). A salicylic acid-induced rice (*Oryza sativa* L.) transcription factor OsWRKY77 is involved in disease resistance of *Arabidopsis thaliana*. *Plant Biol.* 15 452–461. 10.1111/j.1438-8677.2012.00664.x 23061987

[B17] Lang Y. Zhang Z. Gu X. Yang J. Zhu Q. (2004). Physiological and ecological effects of crimpy leaf character in rice (*Oryza sativa* L.) I. Leaf orientation, canopy structure and light distribution. *Acta Agron. Sin.* 30 806–810.

[B18] Li L. Shi Z. Li L. Shen G. Wang X. An L. (2010). Overexpression of ACL1 (*abaxially curled leaf 1*) increased bulliform cells and induced abaxial curling of leaf blades in rice. *Mol. Plant.* 3 807–817. 10.1093/mp/ssq022 20494951

[B19] Lin Z. Zhan X. Cheng S. Cao L. (2013). Progress on mapping and cloning genes for leaf architecture in rice (*Oryza sativa*. L). *J. Nucl. Agric. Sci.* 27 1662–1669.

[B20] Liu X. Li M. Liu K. Tang D. Sun M. Li Y. (2016). Semi-Rolled Leaf2 modulates rice leaf rolling by regulating abaxial side cell differentiation. *J. Exp. Bot.* 67 2139–2150. 10.1093/jxb/erw029 26873975 PMC4809286

[B21] Micol J. L. Hake S. (2003). The development of plant leaves. *Plant Physiol.* 131 389–394. 10.1104/pp.015347 12586863 PMC1540281

[B22] Moffett P. Farnham G. Peart J. Baulcombe D. C. (2002). Interaction between domains of a plant NBS-LRR protein in disease resistance-related cell death. *EMBO J.* 21 4511–4519. 10.1093/emboj/cdf453 12198153 PMC126192

[B23] Molendijk A. J. Ruperti B. Singh M. K. Dovzhenko A. Ditengou F. A. Milia M. (2008). A cysteine-rich receptor-like kinase NCRK and a pathogen-induced protein kinase RBK1 are Rop GTPase interactors. *Plant J.* 53 909–923. 10.1111/j.1365-313X.2007.03384.x 18088316

[B24] Ray D. K. Ramankutty N. Mueller N. D. West P. C. Foley J. A. (2012). Recent patterns of crop yield growth and stagnation. *Nat Commun.* 3 1293. 10.1038/ncomms2296 23250423

[B25] Schoonbeek H. Wang H. Stefanato F. L. Craze M. Bowden S. Wallington E. (2015). Arabidopsis EF-Tu receptor enhances bacterial disease resistance in transgenic wheat. *New Phytol.* 206 606–613. 10.1111/nph.13356 25760815

[B26] Shi Z. Wang J. Wan X. Shen G. Wang X. Zhang J. (2007). Over-expression of rice OsAGO7 gene induces upward curling of the leaf blade that enhanced erect-leaf habit. *Planta* 226 99–108. 10.1007/s00425-006-0472-0 17216479

[B27] Shin B. Choi G. Yi H. K. Yang S. C. Cho I. S. Kim J. (2002). AtMYB21, a gene encoding a flower-specific transcription factor, is regulated by COP1. *Plant J.* 30 23–32. 10.1046/j.1365-313X.2002.01264.x 11967090

[B28] Shinya T. Yamaguchi K. Desaki Y. Yamada K. Narisawa T. Kobayashi Y. (2014). Selective regulation of the chitin-induced defense response by the Arabidopsis receptor-like cytoplasmic kinase PBL27. *Plant J.* 79 56–66. 10.1111/tpj.12535 24750441

[B29] Shiu S. H. Bleecker A. B. (2001). Plant receptor-like kinase gene family: diversity, function, and signaling. *Sci. STKE* 2001:re22. 10.1126/stke.2001.113.re22 11752632

[B30] Shiu S. H. Karlowski W. M. Pan R. Tzeng Y. H. Mayer K. F. Li W. H. (2004). Comparative analysis of the receptor-like kinase family in Arabidopsis and rice. *Plant Cell* 16 1220–1234. 10.1105/tpc.020834 15105442 PMC423211

[B31] Sinclair T. R. Sheehy J. E. (1999). Erect leaves and photosynthesis in rice. *Science* 283 1456–1457. 10.1126/science.283.5407.1455c10206873

[B32] Sturn A. Quackenbush J. Trajanoski Z. (2002). Genesis: cluster analysis of microarray data. *Bioinformatics* 18 207–208. 10.1093/bioinformatics/18.1.207 11836235

[B33] Sugimoto K. Takeda S. Hirochika H. (2000). MYB-related transcription factor NtMYB2 induced by wounding and elicitors is a regulator of the tobacco retrotransposon *Tto1* and defense-related genes. *Plant Cell* 12 2511–2527. 10.1105/tpc.12.12.2511 11148294 PMC102234

[B34] Thimm O. Blasing O. Gibon Y. Nagel A. Meyer S. Kruger P. (2004). MAPMAN: a user-driven tool to display genomics data sets onto diagrams of metabolic pathways and other biological processes. *Plant J.* 37 914–939. 10.1111/j.1365-313X.2004.02016.x 14996223

[B35] Thompson J. D. Gibson T. J. Plewniak F. Jeanmougin F. Higgins D. G. (1997). The CLUSTAL_X windows interface: flexible strategies for multiple sequence alignment aided by quality analysis tools. *Nucleic Acids Res.* 25 4876–4882. 10.1093/nar/25.24.4876 9396791 PMC147148

[B36] Trapnell C. Williams B. A. Pertea G. Mortazavi A. Kwan G. van Baren M. J. (2010). Transcript assembly and quantification by RNA-Seq reveals unannotated transcripts and isoform switching during cell differentiation. *Nat. Biotechnol.* 28 511–515. 10.1038/nbt.1621 20436464 PMC3146043

[B37] Veronese P. Nakagami H. Bluhm B. Abuqamar S. Chen X. Salmeron J. (2006). The membrane-anchored *BOTRYTIS-INDUCED KINASE1* plays distinct roles in *Arabidopsis* resistance to necrotrophic and biotrophic pathogens. *Plant Cell* 18 257–273. 10.1105/tpc.105.035576 16339855 PMC1323497

[B38] Vij S. Giri J. Dansana P. K. Kapoor S. Tyagi A. K. (2008). The receptor-like cytoplasmic kinase (*OsRLCK*) gene family in rice: organization, phylogenetic relationship, and expression during development and stress. *Mol. Plant* 1 732–750. 10.1093/mp/ssn047 19825577

[B39] Walling L. L. Thompson G. A. (2012). “Behavioral and molecular-genetic basis of resistance against phloem-feeding insects,” in *Phloem: Molecular Cell Biology, Systemic Communication, Biotic Interactions*, eds Thompson G. A. van Bel A. J. E. (Oxford: Wiley-Blackwell), 328–351. 10.1002/9781118382806.ch16

[B40] Wang D. Guo Y. Wu C. Yang G. Li Y. Zheng C. (2008). Genome-wide analysis of CCCH zinc finger family in *Arabidopsis* and rice. *BMC Genomics* 9:44. 10.1186/1471-2164-9-44 18221561 PMC2267713

[B41] Wang J. Wu G. Peng C. Zhou X. Li W. He M. (2016). The receptor-like cytoplasmic kinase OsRLCK102 regulates XA21-mediated immunity and plant development in rice. *Plant Mol. Biol. Rep.* 34 628–637. 10.1007/s11105-015-0951-1

[B42] Wang Y. Guo H. Li H. Zhang H. Miao X. (2012). Identification of transcription factors potential related to brown planthopper resistance in rice via microarray expression profiling. *BMC Genomics* 13:687. 10.1186/1471-2164-13-687 23228240 PMC3538557

[B43] Wang Z. Gerstein M. Snyder M. (2009). RNA-Seq: a revolutionary tool for transcriptomics. *Nat. Rev. Genet.* 10 57–63. 10.1038/nrg2484 19015660 PMC2949280

[B44] Wei Z. Hu W. Lin Q. Cheng X. Tong M. Zhu L. (2009). Understanding rice plant resistance to the brown planthopper (*Nilaparvata lugens*): a proteomic approach. *Proteomics* 9 2798–2808. 10.1002/pmic.200800840 19405033

[B45] Xiang J. Zhang G. Qian Q. Xue H. (2012). *SEMI-ROLLED LEAF1* encodes a putative glycosylphosphatidylinositol-anchored protein and modulates rice leaf rolling by regulating the formation of bulliform cells. *Plant Physiol.* 159 1488–1500. 10.1104/pp.112.199968 22715111 PMC3425193

[B46] Xu Y. Wang Y. Long Q. Huang J. Wang Y. Zhou K. (2014). Overexpression of *OsZHD1*, a zinc finger homeodomain class homeobox transcription factor, induces abaxially curled and drooping leaf in rice. *Planta* 239 803–816. 10.1007/s00425-013-2009-7 24385091

[B47] Yamaguchi K. Yamada K. Ishikawa K. Yoshimura S. Hayashi N. Uchihashi K. (2013). A receptor-like cytoplasmic kinase targeted by a plant pathogen effector is directly phosphorylated by the chitin receptor and mediates rice immunity. *Cell Host Microbe.* 13 347–357. 10.1016/j.chom.2013.02.007 23498959

[B48] Yuan L. P. (1997). Super-high yield hybrid rice breeding. *Hybrid Rice* 12 1–6.

[B49] Zhang G. Xu Q. Zhu X. Qian Q. Xue H. (2009). SHALLOT-LIKE1 is a KANADI transcription factor that modulates rice leaf rolling by regulating leaf abaxial cell development. *Plant Cell* 21 719–735. 10.1105/tpc.108.061457 19304938 PMC2671703

[B50] Zhang J. Wu S. Jiang L. Wang J. Zhang X. Guo X. (2015). A detailed analysis of the leaf rolling mutant *sll2* reveals complex nature in regulation of bulliform cell development in rice (*Oryza sativa* L.). *Plant Biol.* 17 437–448. 10.1111/plb.12255 25213398

[B51] Zhao Y. Huang J. Wang Z. Jing S. Wang Y. Ouyang Y. (2016). Allelic diversity in an NLR gene BPH9 enables rice to combat planthopper variation. *Proc. Natl. Acad. Sci. U.S.A.* 113 12850–12855. 10.1073/pnas.1614862113 27791169 PMC5111712

[B52] Zhou X. Wang J. Peng C. Zhu X. Yin J. Li W. (2016). Four receptor-like cytoplasmic kinases regulate development and immunity in rice. *Plant Cell Environ.* 39 1381–1392. 10.1111/pce.12696 26679011

[B53] Zou L. Sun X. Zhang Z. Liu P. Wu J. Tian C. (2011). Leaf rolling controlled by the homeodomain leucine zipper class IV gene *Roc5* in rice. *Plant Physiol.* 156 1589–1602. 10.1104/pp.111.176016 21596949 PMC3135938

