# Supplementary material for: Overexpression of OsRRK1 Changes Leaf Morphology and Defense to Insect in Rice
Source: Front Plant Sci. 2017 Oct 24;8:1783. doi: 10.3389/fpls.2017.01783 (PMC5660730; doi:10.3389/fpls.2017.01783)
Supplement: Supplementary file 1 [file Table_1.pdf]

**Table S1** Primer sequences used in these experiments

| Experiment | Accession no. | Forward (5'-3') | Reverse (5'-3') |
|---|---|---|---|
| Y2H construct | AD-OslecRK | CGGAATTCATGGTTGCTCTGCTACTCTTT | CGGAATTCTTAATGAGAAAACAGAGGATTACATTCC |
| | BD-OsRRK1 | CGGAATTCATGAGGCCTCTGTACCTGCGCAGC | CGGAATTCCTAATTGCTCAAAGATGATGAGCA |
| Overexpression construct | OE-OsRRK1 | ATGAGGCCTCTGTACCTGC | CTAATTGCTCAAAGATGATGAGC |
| Co-IP | Co-LecRK | ATGGTTGCTCTGCTACTCTTT | ATGAGAAAACAGAGGATTACA |
| | Co- RRK1 | ATGAGGCCTCTGTACCTGCGCAGC | ATTGCTCAAAGATGATGAGCAAG |
| RNAi construct | RNAi-OsRRK1-1 | GGATCATCCACAGGGACATC | GGGGTACCGAATTCCTCGAGTCTCCATAGCTCCTCGTCGT |
| | RNAi-OsRRK1-2 | GGATCATCCACAGGGACATC | CAGTTGGGAAATTGGGTTCGAATCTCCATAGCTCCTCGTCGT |
| | PDK | CTCGAGGAATTCGGTACCCC | TTCGAACCCAATTTCCCAACTG |
| probe | hyg | GCTCCATACAAGCCAACCAC | GAAAAAGCCTGAACTCACCG |
| qRT-PCR | OsRRK1 | CCGGCAAGAACGGTGGACGA | TGAGCAAGAAGATGGGGATG |
| | OsRLCK167 | CCTCGTCCTTCCCTCTCGTG | CTGCTGCTGCTGTCTGTCTC |
| | LOC_Os07g04110 | CTTGCTGCTTCTACTTCTTG | CTACGCCGTCGAGGGTGAGG |
| | LOC_Os08g14950 | TGCCCTCCTCTTGCTCACAG | CCCTCTTGGGTCGTTTCTTA |
| | LOC_Os11g31540 | CAAAGGCTGGCATGGAAGGA | GCGGACGACGGAGTTGTTAT |
| | LOC_Os11g40480 | AGGAAATATCCCAGATAGCA | AGGCCAAGATAAGTCAAAGT |
| | LOC_Os01g40260 | CTGTGTCCAGCTACCTCTCC | TGAAGAGAGCGATCACCTC |
| | LOC_Os02g49840 | GGGGAAGATAGTGATAAGGA | GTGCTGGAGAAGACGACAA |
| | LOC_Os11g17954 | TGTTACAACTGCCTACAATA | CATAGATAGATTTCACCTCC |
| | LOC_Os12g24490 | GCTCAGCCAGGAGGACATCG | CGTGTCCCCGTCCTTCACG |
| | *ACL1* | CTGAAGCTGAACCTCTCGCTG | GGAGCATGACGTAGATGAAGCAG |
| | *SRL1* | CCTTCACCTCTCCAATGT | AGGCATCGTTGATAGCAT |
| | *SLL1* | CAGCTCGCAGGTGTCCAA | CCTCCCTAGAGTGAACTCGAGACT |
| | *RL14* | CTCTTTCAGGCATTCCATTGATG | CAACACCTTGTCAGCTTTCAAGC |
| | Actin | GATCACTGCCTTGGCTCCTA | GTACTCAGCCTTGGCAATCC |

**Table S2** Summary of read numbers based on the RNA-Seq data from different rice plants

| Sample ID | Raw reads | Clean reads | Clean ratio | Mapping reads | Mapping ratio |
|---|---|---|---|---|---|
| H1 | 20,771,423 | 20,309,854 | 97.78% | 19,801,822 | 97.50% |
| H2 | 19,498,183 | 19,060,611 | 97.76% | 18,610,233 | 97.64% |
| H3 | 19,126,495 | 18,703,314 | 97.79% | 18,260,886 | 97.63% |
| OE1 | 21,489,449 | 21,010,064 | 97.77% | 20,494,892 | 97.55% |
| OE2 | 18,552,715 | 18,136,501 | 97.76% | 17,654,287 | 97.34% |
| OE3 | 16,995,634 | 16,618,327 | 97.78% | 16,220,304 | 97.60% |
| all | 116,433,899 | 113,838,671 | | 111,042,424 | |

**Table S3** Gene expression of the transcriptome

| | | gene_id | H1_FPKM | H2_FPKM | H3_FPKM | OE1_FPKM | OE2_FPKM | OE3_FPKM | P-value |
|---|---|---|---|---|---|---|---|---|---|
| Kinase | LecRK V.9 | LOC_Os07g04110 | 0.717403 | 0.746349 | 0.603173 | 0.983514 | 1.73309 | 1.70705 | 0.0346 |
| | EFR | LOC_Os08g14950 | 0.0752372 | 0.0647552 | 0.0161649 | 0.463893 | 0.64374 | 0.299835 | 0.0145 |
| | SERK1 | LOC_Os11g31540 | 3.88309 | 3.31006 | 4.51311 | 8.8522 | 11.8752 | 11.3489 | 0.0024 |
| | At3g21340 | LOC_Os11g40480 | 0.0677175 | 0.289006 | 0.153304 | 0.270969 | 0.24414 | 0.0826403 | 0.7543 |
| TFs | WRKY77 | LOC_Os01g40260 | 6.62125 | 5.74177 | 9.56843 | 9.77399 | 9.71762 | 8.4111 | 0.1837 |
| | MADS57 | LOC_Os02g49840 | 0.820737 | 0.938939 | 0.879107 | 8.03309 | 9.32915 | 6.7093 | 0.0109 |
| | MYB | LOC_Os11g17954 | 0.101891 | 0.163609 | 0.109493 | 3.53196 | 4.19536 | 4.15072 | 0.0029 |
| | ZF | LOC_Os12g24490 | 2.03087 | 1.76976 | 2.72508 | 4.83062 | 4.84656 | 4.49034 | 0.0012 |
| leaf rolling-related | REL1 | LOC_Os01g64380 | 48.2414 | 43.7155 | 46.1814 | 48.1709 | 52.4942 | 63.2951 | 0.1400 |
| | LC2 | LOC_Os02g05840 | 16.6921 | 18.638 | 17.3591 | 17.8908 | 19.0833 | 18.4997 | 0.2363 |
| | Roc5 | LOC_Os02g45250 | 36.2094 | 32.6327 | 32.7152 | 33.8904 | 31.7833 | 34.8439 | 0.8270 |
| | ADL1 | LOC_Os02g47970 | 15.5037 | 16.2817 | 17.2914 | 15.7161 | 18.0115 | 16.0866 | 0.7938 |
| | COW1/NAL7 | LOC_Os03g06654 | 1.58158 | 1.77082 | 1.90683 | 1.63895 | 2.01889 | 1.89913 | 0.5354 |
| | ACL1 | LOC_Os04g33860 | 16.8051 | 18.4465 | 16.1292 | 17.4625 | 17.0845 | 15.6277 | 0.6738 |
| | SRL1 | LOC_Os07g01240 | 18.9061 | 18.9869 | 18.9963 | 17.2659 | 19.2873 | 19.5094 | 0.7367 |
| | SLL2 | LOC_Os07g38664 | 10.9338 | 10.39 | 11.1319 | 9.02904 | 8.38372 | 11.3846 | 0.2637 |
| | OsMYB103L | LOC_Os08g05520 | 6.40308 | 6.80692 | 5.41798 | 4.83605 | 5.43255 | 6.85252 | 0.5274 |
| | SLL1 | LOC_Os09g23200 | 4.60338 | 3.63594 | 4.41174 | 3.6579 | 4.48301 | 4.38471 | 0.9206 |
| | OsZHD1 | LOC_Os09g29130 | 2.30522 | 2.25831 | 3.11501 | 1.59935 | 1.47911 | 2.94624 | 0.3700 |
| | RL14 | LOC_Os10g40960 | 13.5199 | 13.6241 | 13.2778 | 13.3935 | 12.1913 | 10.0453 | 0.2436 |
| | NRL1 | LOC_Os12g36890 | 5.72736 | 4.37677 | 6.88933 | 3.67764 | 4.1466 | 6.41129 | 0.4553 |
