# Supplementary material for: Overexpression of OsRRK1 Changes Leaf Morphology and Defense to Insect in Rice
Source: Front Plant Sci. 2017 Oct 24;8:1783. doi: 10.3389/fpls.2017.01783 (PMC5660730; doi:10.3389/fpls.2017.01783)
Supplement: Supplementary file 2 [file Image_1.pdf]

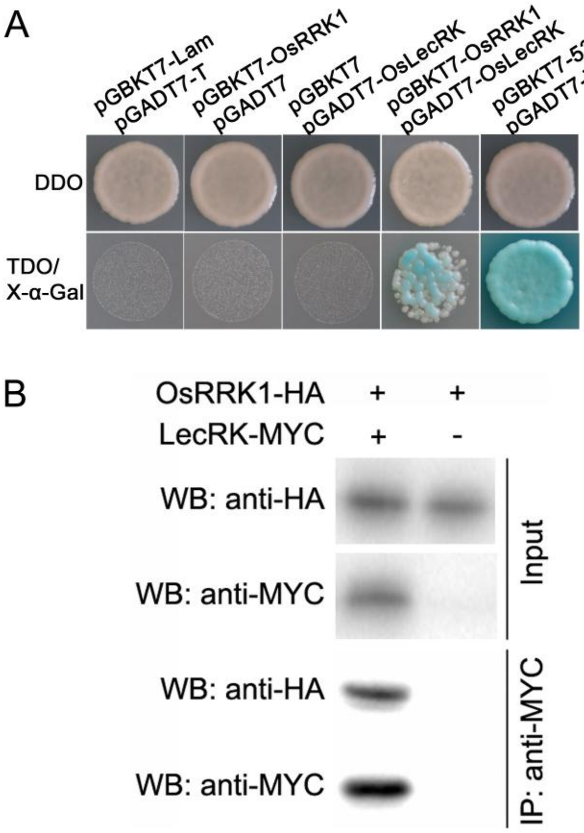

Figure S1. OsRRK1 interacts with OsLecRK. (A)Yeast two-hybrid assay of the interaction between OsRRK1 and OsLecRK. The diploids were grown on DDO (SD/-Leu/-Trp) plates and transferred to TDO (SD/-Leu/-Trp/-His) plates with 20 μg mL^{-1} X-α-Gal. SD medium is defined as the minimal media that is used for culturing yeast cells including carbon and essential amino acids. (B) CoIP of the interaction between OsRRK1 and OsLecRK *in vivo*. OsRRK1 were fused to a HA tag, and OsLecRK was fused to a Myc tag. The OsRRK1:HA and OsLecRK:Myc constructs were expressed in rice protoplasts. Co-immunoprecipitation was performed using the anti-HA antibody used to detect the OsRRK1, and the precipitated protein was analyzed by Western blotting using an anti-Myc antibody to detect OsLecRK.

```
1     GTGCGTATCCCCGCGAAACGCCTCGTCGCGTTCATATTTGCTCACGGCTCGCGCGGCACTCGCGAGAGCACCTCGGCGTTAGTACCCATC

91    AATGCCTCCCTCCTCCTCCTCCTCCGCCACCACCACCAGCTCGCCGTCTCGTCTCCCTCCTCCGCTTCGCCTCCTCTGATCACCTCGCTC

181   GCCGGCGACGCCGCGATCGCCACCGCACCGCAGGGCATTGTGTCGTCGAGGGGAGAGATGAGGCCTCTGTACCTGCGCAGCAGCGGCAGC
                                                                M  R  P  L  Y  L  R  S  S  G  S

271   TTCAAGAAGCTGCTCCTCTCCATCAGCCACCGCGGCGCCAAGAACGGCAATGGCGACGCCGCCGCCAAGGAGAGATACACGCCCGCCGCC
       F  K  K  L  L  L  S  I  S  H  R  G  A  K  N  G  N  G  D  A  A  A  K  E  R  Y  T  P  A  A

361   GCCGCGGCGGCGCCGGAGTCGCCGCGGAAGCCGGCGTGGCGGTGCTTCTCCTACGAGGAGATCCACCGCGCCACGAACGCGTTCCACGAG
       A  A  A  A  P  E  S  P  R  K  P  A  W  R  C  F  S  Y  E  E  I  H  R  A  T  N  A  F  H  E

451   GGCAACCTGGTGGGGAAGGGCGGGTCGTCGGAGGTGTACCGCGGCGAGCTCCCCGACGGGCGGGCCGTGGCGGTGAAGCGGCTGATGGGC
       G  N  L  V  G  K  G  G  S  S  E  V  Y  R  G  E  L  P  D  G  R  A  V  A  V  K  R  L  M  G

541   GCGTGGGCGTGCGAGCGGCGGGAGCGCGACTTCCTGGCGGAGCTCGGCACGGTGGGGCACGCGCGCCACCCCAACGTGTGCGCCCTCCTC
       A  W  A  C  E  R  R  E  R  D  F  L  A  E  L  G  T  V  G  H  A  R  H  P  N  V  C  A  L  L

631   GGCTGCTGCGTCGACCGCGACCTCTACCTCGTCTTCCACTTCTCCGGCCGCGGCTCCGTCTCCGCCAACCTCCACGACGAGAAGAAGGCG
       G  C  C  V  D  R  D  L  Y  L  V  F  H  F  S  G  R  G  S  V  S  A  N  L  H  D  E  K  K  A

721   CCGGCGATGGGGTGGGCGGTGCGGCGCGCCATTGCCGTCGGCACGGCGAGGGGGCTGGAGTACCTGCACAAGGGGTGCCAGAGGAGGATC
       P  A  M  G  W  A  V  R  R  A  I  A  V  G  T  A  R  G  L  E  Y  L  H  K  G  C  Q  R  R  I

811   ATCCACAGGGACATCAAGGCATCCAACGTGCTTCTCACCGACGACTTCCAGCCTCAGATTTCCGATTTCGGGCTCGCCAAGTGGCTGCCG
       I  H  R  D  I  K  A  S  N  V  L  L  T  D  D  F  Q  P  Q  I  S  D  F  G  L  A  K  W  L  P

901   TCGGAGTGGACGCACCGGGCGATCGCCCCGATCGAAGGAACATTCGGGTGTTTGGCACCGGAGTACTACACGCATGGCATCGTCGACGAG
       S  E  W  T  H  R  A  I  A  P  I  E  G  T  F  G  C  L  A  P  E  Y  Y  T  H  G  I  V  D  E

991   AAGACGGACGTGTTCGCGTTCGGCGTCTTCCTCCTGGAGATCATGACCGGGAGAAAGCCGGTGGATGGCAGCCACAAGAGCTTGCTCAGC
       K  T  D  V  F  A  F  G  V  F  L  L  E  I  M  T  G  R  K  P  V  D  G  S  H  K  S  L  L  S

1081  TGGGCGAGGCCGTTCCTGAACGAGGGGAGGATCGAGTCGCTGGTGGATCCGAGGATCGGCGGCGACTACGACGGCGAGGAGGCGCGGCGG
       W  A  R  P  F  L  N  E  G  R  I  E  S  L  V  D  P  R  I  G  G  D  Y  D  G  E  E  A  R  R

1171  CTGGCGTTCGTGGCGTCGCTGTGCATCCGCTCGTCGGCGAAGTGGAGGCCATCCATGACCGAGGTGCTGGAGCTGCTGGAAGGCGTCGAG
       L  A  F  V  A  S  L  C  I  R  S  S  A  K  W  R  P  S  M  T  E  V  L  E  L  L  E  G  V  E

1261  ATCCGGCAAGAACGGTGGACGATGCCCGAGGCAGTGGAGGACGACGACGACGAGGAGCTATGGAGATTCGATGACCTGGATGACGAAGAC
       I  R  Q  E  R  W  T  M  P  E  A  V  E  D  D  D  D  E  E  L  W  R  F  D  D  L  D  D  E  D

1351  GACGAAGATGAAGAGGAGTTCAACACTGCATCCCCATCTTCTTGCTCATCATCTTTGAGCAATTAGTAGTTAGTAATAGTATCTAAGATT
       D  E  D  E  E  E  F  N  T  A  S  P  S  S  C  S  S  S  L  S  N  *

1441  GTGAGTAAGCTTGTGATGTTGTTACAAGATGAAATTTTTGATAGTTACTCTCCACTGGGCCCACTTGTAAATAGAAAGGATTTTCATCTT

1531  ATCATCATCCAACAGGCTATGAAAAGCAAGTAGTACC
```

Figure S2. Full-length cDNA of OsRRK1 and its deduced amino acid residues. The red letters represent a conserved domain.

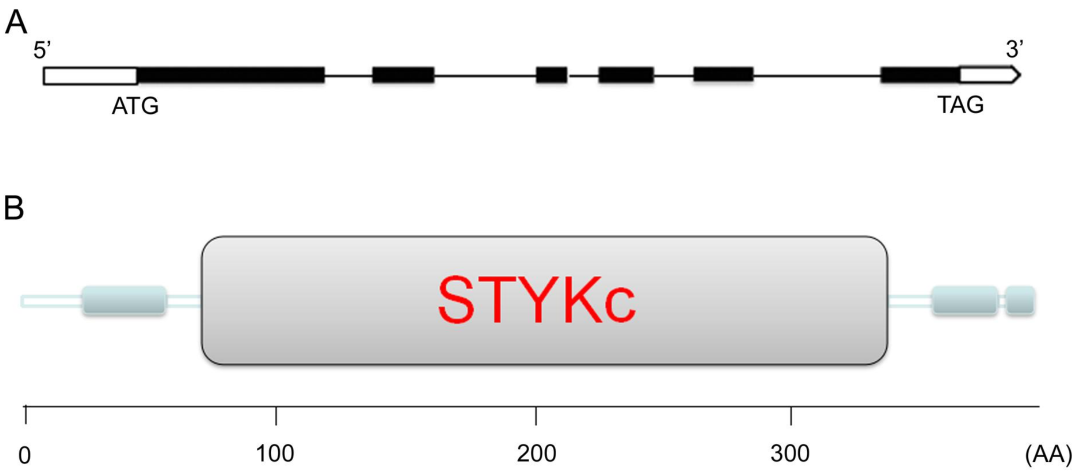

Figure S3. Gene structure (A) and protein structure (B) of OsRRK1. (A) *OsRRK1* comprises six exons and five introns. The white frames represent the 5'UTR and 3' UTR, the black frame represents exons, the line represents introns. (B) *OsRRK1* encodes a polypeptide of 392 amino acids with a Serine-threonine/tyrosine-protein kinase catalytic (STYKc) domain.

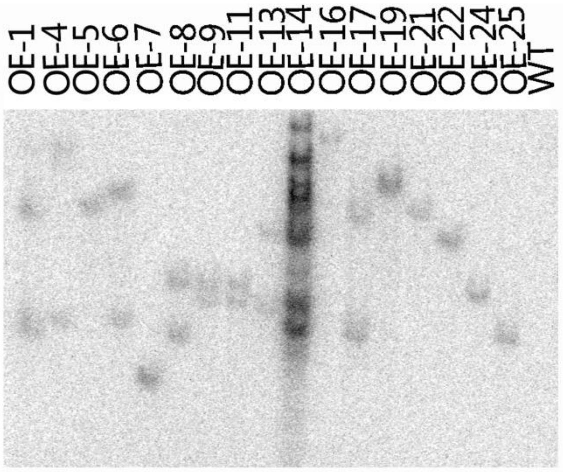

Figure S4. Southern blot analysis of OE-*OsRRK1* transgenic plants. OE-22, OE-24 and OE-25 were single copies of *OsRRK1* plants.

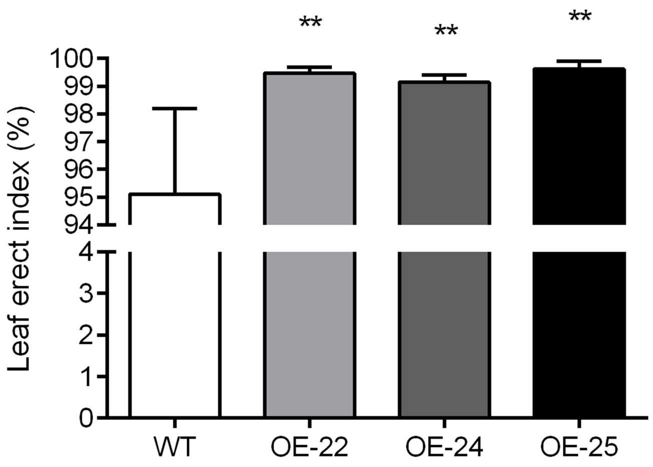

Figure S5. LEI of the flag leaves of the WT and OE-*OsRRK1* plants. Error bars indicate SD (n=20). The significances are indicated by the asterisks: *, Student's t-test, $P < 0.05$; **, Student's t-test, $P < 0.01$.

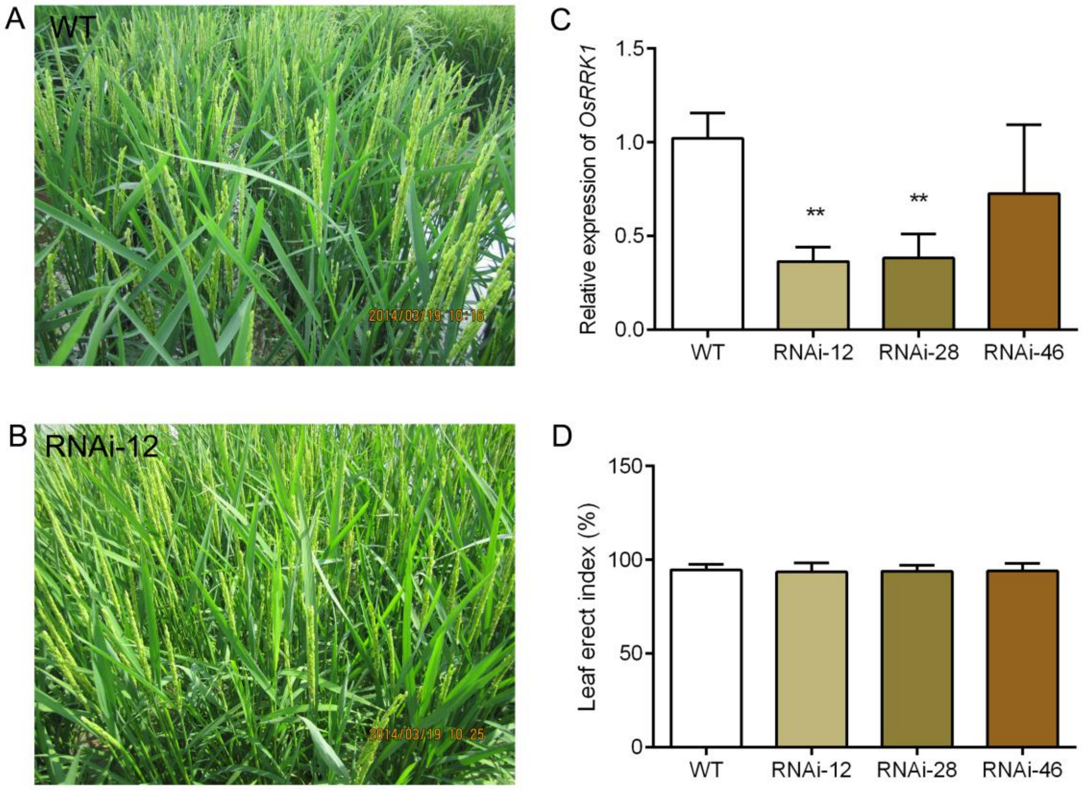

Figure S6. Phenotypes of the WT and the RNAi-*OsRRK1* plants. (A-B) Morphology of WT plants (A) and the RNAi-12 (B) in the paddy field. (C) The relative expression levels of *OsRRK1* in the WT and RNAi-*OsRRK1* plants. (D) LEI of the flag leaves of the WT and RNAi-*OsRRK1* plants at the heading stage. *, Student's t-test, $P < 0.05$; **, Student's t-test, $P < 0.01$.

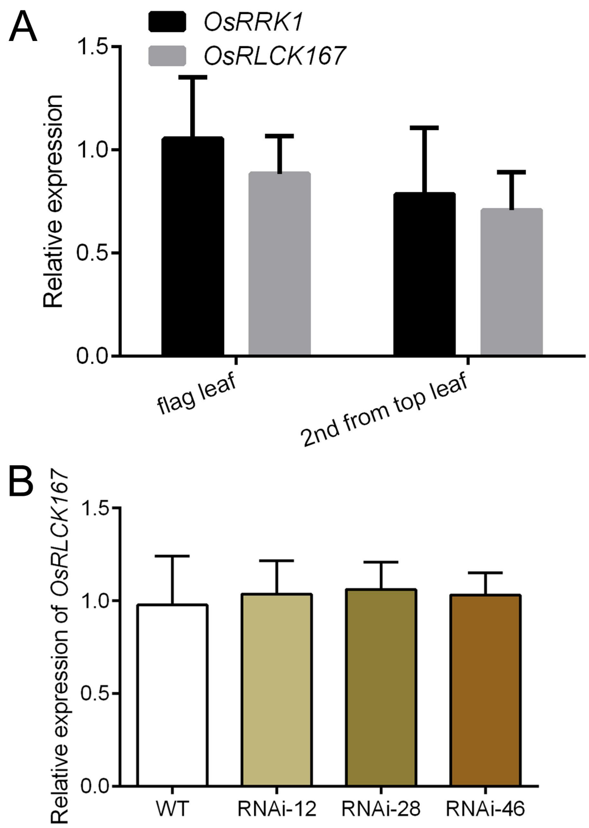

Figure S7. The relative expression levels of OsRRK1 and OsRLCK167. (A) The relative expression levels of OsRRK1 and OsRLCK167 in the leaf at heading stage of WT plants. (B) The relative expression levels of OsRLCK167 in the RNAi-OsRRK1 plants.

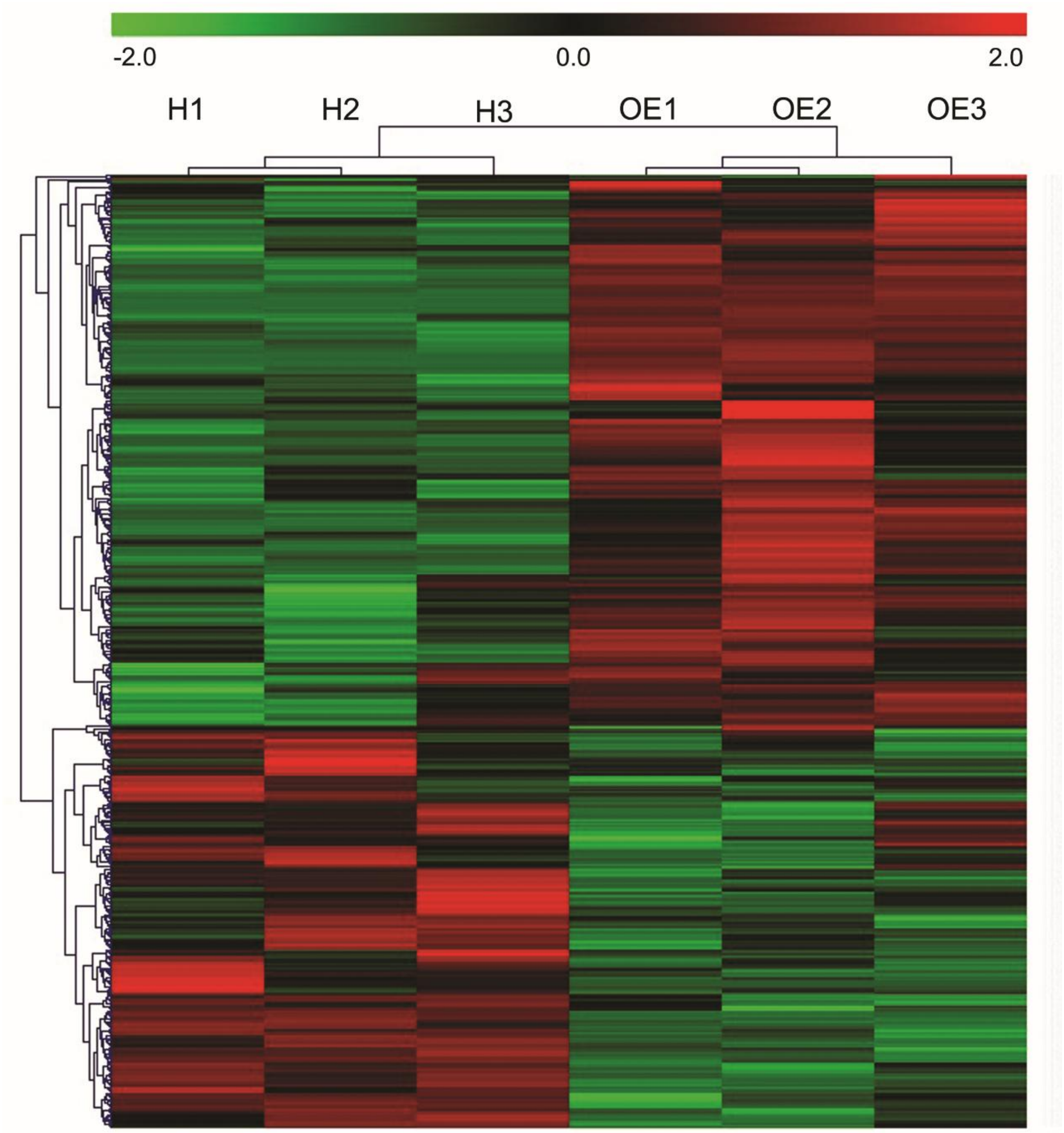

Figure S8. Hierarchical clustering analysis of DEGs based on log ratio of FPKM data. The color key represents FPKM normalized log_2 transformed counts. Red represents up-regulated DEGs, green represents down-regulated DEGs. Each column represents a comparison group, each row represents a gene.
